# Joint Dwell Time and Bandwidth Optimization for Multi-Target Tracking in Radar Network Based on Low Probability of Intercept

**DOI:** 10.3390/s20051269

**Published:** 2020-02-26

**Authors:** Lintao Ding, Chenguang Shi, Wei Qiu, Jianjiang Zhou

**Affiliations:** 1 Key Laboratory of Radar Imaging and Microwave Photonics, Ministry of Education, Nanjing University of Aeronautics and Astronautics, Nanjing 210016, China; dltnuaa@163.com (L.D.); 15250956004@163.com (W.Q.); zjjee@nuaa.edu.cn (J.Z.); 2 Science and Technology on Electro-Optic Control Laboratory, Luoyang 471009, China

**Keywords:** low probability of intercept (LPI), Bayesian Cramer–Rao lower bound (BCRLB), multi-target tracking, radar network

## Abstract

Radar network systems have been demonstrated to offer numerous advantages for target tracking. In this paper, a low probability of intercept (LPI)-based joint dwell time and bandwidth optimization strategy is proposed for multi-target tracking in a radar network. Since the Bayesian Cramer–Rao lower bound (BCRLB) provides a lower bound on parameter estimation, it can be utilized as the accuracy metric for target tracking. In this strategy, in order to improve the LPI performance of the radar network, the total dwell time consumption of the underlying system is minimized, while guaranteeing a predetermined tracking accuracy. There are two adaptable parameters in the optimization problem: one for dwell time, and the other for bandwidth allocation. Since the nonlinear programming-based genetic algorithm (NPGA) can solve the nonlinear problem well, we develop a method based upon NPGA to solve the resulting problem. The simulation results demonstrate that the proposed strategy has superiority over traditional algorithms, and can achieve a better LPI performance of this radar network.

## 1. Introduction

Recently, radar network systems, such as multiple-input multiple-output (MIMO) radar, have attracted great attention from academic researchers [1,2,3,4,5]. It has been shown that a radar network system has numerous potential advantages over traditional monostatic and bistatic radar, such as waveform diversity [1], multiplexing gain [2], enhanced target tracking, localization performance [6,7], etc. As far as multi-target tracking in a radar network, in order to best utilize the system potential under the limited system resources, the resource allocation is of great importance, and receives more and more attention in recent years [8,9,10,11,12,13,14,15,16,17,18,19,20,21].

An effective radar resource allocation strategy can efficiently optimize system parameters, leading to performance enhancements. Therefore, it is necessary to allocate the total launch resources in the radar networks reasonably. As we all know, power allocation is one crucial factor in the resource management of any radar network [8,9,10,11]. Godrich et al. (2011) [9] proposed a power allocation strategy for target localization in distributed MIMO radar systems, whose objective can be divided into two parts. In the first part, the total transmission power is minimized for a given accuracy requirement, while in the latter part, the tracking accuracy is maximized under the constraint of a given power budget. As an extension, Xie et al. [10] extended this work to a more general case of unknown previous position information, which promotes the real-time applications.

A performance-driven power allocation algorithm is proposed by maximizing the achievable tracking accuracy with a given total power budget [11]. The algorithm can be regarded as the response of the cognitive transmitter to the environment, which is observed by the receiver in the radar network.

In addition, the time resource allocation is also critical, such as revisit time and dwell time allocation [12,13,14]. The concept of radar dwell time optimization for target tracking is studied for the first time [12], under the premise of meeting the predetermined target tracking accuracy requirements, and the total dwell time of the phased array radars is minimized. Narykov et al. [13] employed the Markov decision to manage the time resource for target tracking. Specifically, the dwell time and revisit time are adjusted adaptively to increase the maximum number of tracking targets. Wang et al. [14] proposed a joint revisit and dwell time management strategy for single target tracking, which aims to minimize the total time resource used for target tracking, while meeting a desired tracking accuracy requirement.

However, most of the above researches only focus on the single parameter optimization. On the basis of the research mentioned above, many joint resource management optimization algorithms are proposed. Yan et al. [15] proposed a joint beam selection and power allocation strategy for multiple targets tracking, whose basis is to allocate the limited beam and power resource of the radar network for the purpose of achieving an accurate target state estimation. Xie et al. [16] take two variable parameters into consideration: The number of radar nodes and the transmitted power of radar network, and then propose a joint node selection and power allocation strategy with the objective of tracking multiple targets. A cooperative nodes and transmit waveform scheduling scheme is proposed for multiple targets tracking in a distributed radar network [17], where this scheme aims at minimizing the cost of the allocation of waveforms, while guaranteeing a predefined target tracking accuracy.

Although the above works provide us an opportunity to deal with resource management, they have little regard of the low probability of intercept (LPI) performance in radar network systems. With the development of passive detectors, such as the radar warning receiver (RWR), electronic warfare support (ES), anti-radiation missile (ARM), and so on, a serious threat is posed to the radar network. As a result, the study of LPI optimization for radar network systems has attracted significant interest in recent years [18,19,20,21,22,23]. She et al. [21] proposed a sensor selection and power allocation algorithm for multi-target tracking, whose basis is to reduce the total transmitted power under the constraint of target tracking accuracy, with the purpose of improving the LPI performance of the radar network. A joint transmitter selection and resource management strategy based upon LPI is proposed by controlling transmitting resources while meeting a specified target-tracking accuracy requirement [22]. Generally, the above literature have put forward the idea of joint resource management for LPI performance in radar network systems, which lays a foundation for future study.

For multi-target tracking in a radar network, the information from each monostatic component must be gathered to the fusion center for fusion and processing. However, the data processing rate is commonly limited. Therefore, in order to process all the measurement data before the next observation time and feed back to the radar transmitter in time, it is necessary to strictly control the total amount of data, which is related to the bandwidth of transmitted waveform. Furthermore, the target tracking accuracy is also related to the bandwidth of the radar-transmitted signal. Garcia et al. [24] take the signal bandwidth into account for the first time, and propose a joint power and bandwidth allocation (JPBA) method, with the purpose of maximizing the localization accuracy of a single target. Yan et al. [25] extend the JPBA strategy to the target tracking scenario, where signal bandwidth is allocated to meet the real-time processing requirements. To conclude, bandwidth allocation is also one of the critical factors which needs to be considered in the resource management of radar transmission.

However, to the best of our knowledge, the problem of dwell time allocation and bandwidth allocation to realize the LPI performance optimization for multi-target tracking in a radar network, which has never been taken into consideration, needs to be analyzed in detail.

In this paper, an LPI-based joint dwell time and bandwidth allocation optimization strategy in a radar network is proposed. The strategy can adaptively adjust the radar selection, dwell time and signal bandwidth allocation according to the target motion characteristics at each observation moment. As the Bayesian Cramer–Rao lower bound (BCRLB) combines the revisit time, dwell time, target RCS, transmission signal bandwidth and some other variables, it offers insight effect into the parameters on the tracking performance. Consequently, we utilized BCRLB as the accuracy metric for target tracking. For a predefined target tracking accuracy threshold, the resulting problem is minimizing the total dwell time by optimizing the radar selection, dwell time and transmit signal bandwidth. Then, an efficient two-step method is proposed to solve this problem. Finally, two different RCS cases is considered in this paper to verify the superiority of the proposed strategy.

The remainder of this paper is organized as follows. The system model is introduced in Section 2. Section 3 presents the joint dwell time and bandwidth optimization strategy. In Section 3.1 we derive the BCRLB as the performance metric of the target tracking accuracy. Then, the LPI performance optimization problem based on BCRLB is formulated in Section 3.2. A nonlinear programming-based genetic algorithm (NPGA)-based method is proposed to solve this problem in Section 3.3. Simulation results are provided in Section 4. Finally, conclusions are given in Section 5.

## 2. System Model

### 2.1. Target Dynamic Model

Suppose there are Q scattered targets in a two dimensional space. The qth(q=1,2,…,Q) target is initially located at $\left(x_{0}^{q},y_{0}^{q}\right)$, with initial velocity $\left({\dot{x}}_{0}^{q},{\dot{y}}_{0}^{q}\right)$. Assuming that all of the targets move in a uniform linear line, the dynamic model of the target can be described as:

(1) $$X_{k}^{q}=FX_{k-1}^{q}+W^{q}$$

in (1), $X_{k}^{q}={\left[x_{k}^{q},y_{k}^{q},{\dot{x}}_{k}^{q},{\dot{y}}_{k}^{q}\right]}^{T}$ is the state vector of target q at time index k, where $\left(x_{k}^{q},y_{k}^{q}\right)$ and $\left({\dot{x}}_{k}^{q},{\dot{y}}_{k}^{q}\right)$ are the position and velocity of target q at time index k, respectively. F is the target state transition matrix, which can be expressed as:

(2) $$F=\left[\begin{array}{cccc} 1 & 0 & T & 0\\ 0 & 1 & 0 & T\\ 0 & 0 & 1 & 0\\ 0 & 0 & 0 & 1 \end{array}\right]$$

where T denotes the revisit time. The term $W^{q}$ is the process noise of target q, which can be assumed as zero-mean Gaussian noise with a known covariance $Q^{q}$,

(3) $$Q^{q}=\sigma_{q,w}^{2}\left[\begin{array}{cccc} \frac{T^{3}}{3} & 0 & \frac{T^{2}}{2} & 0\\ 0 & \frac{T^{3}}{3} & 0 & \frac{T^{2}}{2}\\ \frac{T^{2}}{2} & 0 & T & 0\\ 0 & \frac{T^{2}}{2} & 0 & T \end{array}\right]$$

where $\sigma_{q,w}^{2}$ denotes the process noise intensity of target q.

### 2.2. Observation Model

Consider a radar network with N two-dimensional phased array radars (PARs) working in space, time and frequency synchronization. In order to simplify the problem, we give some moderate assumptions:

(1) Each radar can only receive its own echo signals;
(2) A single radar tracks at most one target in a revisit period.

The traditional radar network system requires all of the radars in the system to radiate a target at all times. Due to the limitation of spectrum resources, communication resources, energy resources etc., multi-target tracking in a traditional radar network is inefficient. As a result, it is not necessary for all radars to work in a revisit period. Thus, we define a set of binary variables $u_{i,k}^{q}\in \left\{0,1\right\}$ to represent the radar selection index:

(4) $$u_{i,k}^{q}=\{\begin{array}{l} 1,\;\mathrm{if}\;\mathrm{the}\;q\mathrm{th}\;\mathrm{target}\;\mathrm{is}\;\mathrm{tracked}\;\mathrm{by}\;\mathrm{the}\;i\mathrm{th}\;\mathrm{radar}\;\mathrm{at}\;\mathrm{time}\;\mathrm{index}\;k\\ 0,\;\mathrm{otherwise} \end{array}$$

Assuming that all PARs in the radar network are able to extract the distance and angle information from the echo signal, then the measurement equation can be written as:

(5) $$Z_{i,k}^{q}=\{\begin{array}{cc} h_{i}\left(X_{k}^{q}\right)+V_{i,k}^{q},\; & \mathrm{if}\;u_{i,k}^{q}=1\\ 0\;,\; & \mathrm{if}\;u_{i,k}^{q}=0 \end{array}$$

where $Z_{i,k}^{q}$ represents the measured value, and $h_{i}\left(X_{k}^{q}\right)$ is a nonlinear transfer function with the following expression:

(6) $$h_{i}\left(X_{k}^{q}\right)=\left[\begin{array}{c} R_{i,k}^{q}\\ \theta_{i,k}^{q} \end{array}\right]=\left[\begin{array}{c} \sqrt{{\left(x_{k}^{q}-x_{i}\right)}^{2}+{\left(y_{k}^{q}-y_{i}\right)}^{2}}\\ \arctan\left[\frac{y_{k}^{q}-y_{i}}{x_{k}^{q}-x_{i}}\right] \end{array}\right]$$

here, $\left(x_{i},y_{i}\right)$ denotes the ith radar’s position, $R_{i,k}^{q}$ and $\theta_{i,k}^{q}$ are the qth target’s distance and azimuth to radar i. In (5), $V_{i,k}^{q}$ is the measurement noise and can be written as $V_{i,k}^{q}={\left[\Delta R_{i,k}^{q},\Delta \theta_{i,k}^{q}\right]}^{T}$, where $\Delta R_{i,k}^{q}$ and $\Delta \theta_{i,k}^{q}$ are the measurement errors of distance and azimuth, respectively. Assuming that $V_{i,k}^{q}$ is zero-mean Gaussian noise with covariance $G_{i,k}^{q}$, which can given by:

(7) $$G_{i,k}^{q}=\left[\begin{array}{cc} \sigma_{R_{i,k}^{q}}^{2} & 0\\ 0 & \sigma_{\theta_{i,k}^{q}}^{2} \end{array}\right]$$

herein, $\sigma_{R_{i,k}^{q}}^{2}$ and $\sigma_{\theta_{i,k}^{q}}^{2}$ are the mean square estimation error of distance and azimuth, respectively. Both of them are related to the signal-to-noise ratio (SNR) of the echo at the current moment and can be calculated as [26]:

(8) $$\{\begin{array}{l} \sigma_{R_{i,k}^{q}}=\frac{c}{4\pi \beta_{i,k,q}\sqrt{{\mathrm{SNR}}_{i,k}^{q}}}\\ \sigma_{\theta_{i,k}^{q}}=\frac{\sqrt{3}\lambda }{\pi \gamma \sqrt{{\mathrm{SNR}}_{i,k}^{q}}} \end{array}$$

where ${\mathrm{SNR}}_{i,k}^{q}$ denotes the ith radar’ SNR to target q at time index k. The term $c=3\times 10^{8}\;m/s$ is the speed of light, λ and γ are the transmitted wavelength and antenna aperture, respectively. $\beta_{i,q,k}$ is the effective bandwidth of the ith radar’s transmitted waveform to target q.

It can be seen that under the same conditions of other parameters, the higher the $\beta_{i,q,k}$ in (8), the smaller the measurement error of distance. In addition, the amount of radar data samples from the illuminated targets is also related to the transmitted signal bandwidth. Given the oversampling ratio ρ≥1, the ith radar’s sampling frequency on the qth target at time index k is $f_{i,k}^{s}=\rho \beta_{i,q,k}$ [25]. Then, given the observation area V of radar network, the number of the qth target’s from ith radar can be calculated as:

(9) $$N_{i,q,k}=u_{i,k}^{q}\frac{\rho \beta_{i,q,k}}{c}VM$$

From Equation (8), we can conclude that the measurement error of distance and azimuth is inversely proportional to the SNR of the echo. According to the radar equation, if the beams are unbiased with target when the ith radar irradiate target q at time index k, the echo SNR of a single pulse, can be expressed as:

(10) $${\mathrm{SNR}}_{i,q,k}^{s}=\frac{P_{t}G_{t}G_{r}\sigma^{q}\lambda^{2}G_{\mathrm{RP}}}{{\left(4\pi \right)}^{3}kT_{o}B_{r}F_{r}{\left(R_{i}^{q}\right)}^{4}}$$

where $P_{t}$ denotes the transmitted power of radar; $G_{t}$ is the transmit antenna gain; $G_{r}$ is the receive antenna gain; $\sigma^{q}$ is the radar cross section (RCS) of the target q; $G_{\mathrm{RP}}$, $T_{o}$ and $F_{r}$ are the processing gain, noise temperature and noise coefficient of the radar receiver, respectively; k is the Boltzmann constant; $B_{r}$ is the bandwidth of the radar receiver-matched filter, and $R_{i}^{q}$ is the distance from the ith radar to target q.

During the dwell time of a single irradiation to the target, the radar can receive several reflection pulses from the target. Since the radar has known its own emission parameters, all of the target reflections can be accumulated by coherent accumulation technology to improve the SNR of the echo. Suppose $T_{i,q,k}^{d}$ represents the dwell time of the ith radar’s irradiation on target q at time index k, and $T_{r}$ represents the pulse repetition period of radar, then the number of coherent accumulated pulses can be given by:

(11) $$n_{i,q,k}=\frac{T_{i,q,k}^{d}}{T_{r}}$$

Assuming that coherent accumulation is ideal, the SNR obtained after $n_{i,q,k}$ pulses can be written as:

(12) $${\mathrm{SNR}}_{i,q,k}^{\mathrm{CI}}=n_{i,q,k}{\mathrm{SNR}}_{i,q,k}^{s}$$

When there is an angle difference ${\tilde{\alpha }}_{i}^{q}$ between the true azimuth of target q and the beam pointing of the ith radar, the echo SNR after coherent accumulation can be expressed as:

(13) $${\mathrm{SNR}}_{i,k}^{q}={\mathrm{SNR}}_{i,q,k}^{\mathrm{CI}}\exp\left(-4\ln\left(2\right)\frac{{\left({\tilde{\alpha }}_{i}^{q}\right)}^{2}}{\theta_{3\mathrm{dB}}^{2}}\right)$$

where $\theta_{3\mathrm{dB}}$ denotes 3dB antenna beam width. Substitute Equations (10)–(12) into Equation (13), then we can obtain:

(14) $${\mathrm{SNR}}_{i,k}^{q}=\frac{T_{i,q,k}^{d}}{T_{r}}\frac{P_{t}G_{t}G_{r}\sigma^{q}\lambda^{2}G_{\mathrm{RP}}}{{\left(4\pi \right)}^{3}kT_{o}B_{r}F_{r}{\left(R_{i}^{q}\right)}^{4}}\exp\left(-4\ln\left(2\right)\frac{{\left({\tilde{\alpha }}_{i}^{q}\right)}^{2}}{\theta_{3\mathrm{dB}}^{2}}\right)$$

### 2.3. Fusion Center

We assume that the radar network adopts an indirect centralized fusion method. Specifically, each radar illuminates the assigned target, extracts the measurement information from the echo signal, and transmits the distance and azimuth information to the fusion center through a radio frequency (RF) stealth data link for processing. In this system, suppose that the fusion center can make full use of the original measurement data without any loss of information, and thus the fusion results are the optimal. Therefore, the measurement information about the target q at time index k can be formulated as:

(15) $$Z_{k}^{q}=\left[{\left[1,1\right]}^{T}⊗u_{k}^{q}\right]⊙\left[{\left[{\left(R_{k}^{q}\right)}^{T},{\left(\theta_{k}^{q}\right)}^{T}\right]}^{T}+{\left[{\left(\Delta R_{k}^{q}\right)}^{T},{\left(\Delta \theta_{k}^{q}\right)}^{T}\right]}^{T}\right]$$

where $R_{k}^{q}={\left[R_{1,k}^{q},R_{2,k}^{q},\ldots ,R_{N,k}^{q}\right]}^{T}$ and $\theta_{k}^{q}={\left[\theta_{1,k}^{q},\theta_{2,k}^{q},\ldots ,\theta_{N,k}^{q}\right]}^{T}$ denotes the sets of the distance and azimuth measurement parameters of target q at time index k, respectively, $\Delta R_{k}^{q}={\left[\Delta R_{1,k}^{q},\Delta R_{2,k}^{q},\ldots ,\Delta R_{N,k}^{q}\right]}^{T}$ and $\Delta \theta_{k}^{q}={\left[\Delta \theta_{1,k}^{q},\Delta \theta_{2,k}^{q},\ldots ,\Delta \theta_{N,k}^{q}\right]}^{T}$ are the sets of the distance and azimuth measurement parameter errors, respectively. In (15), the term $u_{k}^{q}$ represents the radar allocation index set of target q at time index k, ⊗ is the matrix direct product operation, and ⊙ is the matrix dot product.

It is assumed that the measurement errors of each radar are independent of each other’s, so the qth target’s measurement noise covariance matrix $G_{k}^{q}$ can be given by:

(16) $$G_{k}^{q}=\mathrm{diag}\left\{u_{1,k}^{q}\sigma_{R_{i,k}^{q}}^{2},u_{2,k}^{q}\sigma_{R_{i,k}^{q}}^{2},\ldots ,u_{N,k}^{q}\sigma_{R_{i,k}^{q}}^{2},u_{1,k}^{q}\sigma_{\theta_{i,k}^{q}}^{2},u_{2,k}^{q}\sigma_{\theta_{i,k}^{q}}^{2},\ldots ,u_{N,k}^{q}\sigma_{\theta_{i,k}^{q}}^{2}\right\}$$

where diag{⋅} denotes diagonal matrix. 

Since the fusion center receives the measurement information from all of the radars in the network on each target, the total number of samples that need to be processed can be calculated as follows:

(17) $$N_{k}=\sum_{q=1}^{Q}\sum_{i=1}^{N}N_{i,q,k}$$

## 3. Joint Dwell Time and Bandwidth Optimization Strategy

Dwell time allocation is one of the critical problems to address for LPI performance in a radar network. Under the assumption that the radiation interval is fixed, in order to improve the RF stealth performance, we should minimize the total dwell time in the radar network. However, according to the statement in Section 2.2 and (8), we can get: the reduction of the dwell time will reduce the echo SNR, which will lead to the decrease of detection probability and tracking accuracy. As a result, the purpose of our work is to minimize the total dwell time of the radar network, which is constrained by a predefined accuracy requirement for target tracking. Furthermore, when it comes to the bandwidth of the transmitted waveform, transmitting a larger bandwidth signal means that the system has a higher accuracy of target distance. However, it will increase the workload of the fusion center at the same time, and even make the fusion center unable to process all of the target information within the effective time. Therefore, under the premise of meeting the constraints of target tracking accuracy, data processing capacity and the limited radar resources, we propose a joint dwell time and bandwidth optimization strategy for multi-target tracking with the objective of improving the LPI performance in the radar network.

### 3.1. Performance Metric

The BCRLB provides a lower bound on the mean square error (MSE) of parameter unbiased estimation, and compares to the posterior Cramer–Rao lower bound (PCRLB) [27,28]. In this paper, BCRLB is derived and used as an optimization criterion for the joint dwell time and bandwidth optimization strategy. At time index k, we use the observation vector $Z_{k}^{q}$ to estimate the state of qth target, which can be defined as ${\hat{X}}_{k}^{q}\left(Z_{k}^{q}\right)$, then the MSE of ${\hat{X}}_{k}^{q}\left(Z_{k}^{q}\right)$ satisfies the following equation:

(18) $$E\left\{\left({\hat{X}}_{k}^{q}\left(Z_{k}^{q}\right)-X_{k}^{q}\right)-{\left({\hat{X}}_{k}^{q}\left(Z_{k}^{q}\right)-X_{k}^{q}\right)}^{T}\right\}=C_{k}^{q}\geq J^{-1}\left(X_{k}^{q}\right)$$

where E{•} denotes mathematical expectation, $C_{k}^{q}$ is the qth target’s BCRLB at time index k, and $J\left(X_{k}^{q}\right)$ is the Bayesian information matrix (BIM), which can be written as:

(19) $$J\left(X_{k}^{q}\right)=-E_{X_{k}^{q},Z_{k}^{q}}\left\{\Delta_{X_{k}^{q}}^{X_{k}^{q}}\log p\left(Z_{k}^{q},X_{k}^{q}\right)\right\}$$

where $\Delta_{X_{k}^{q}}^{X_{k}^{q}}=\nabla_{X_{k}^{q}}\nabla_{X_{k}^{q}}^{T}$, here $\nabla_{X_{k}^{q}}$ denotes the first-order partial derivative vectors. In (19),

(20) $$p\left(Z_{k}^{q},X_{k}^{q}\right)=p\left(X_{k}^{q}\right)p\left(Z_{k}^{q}|X_{k}^{q}\right)$$

is the joint probability density function (PDF) [11].

The BIM $J\left(X_{k}^{q}\right)$ can be expressed as the sum of two matrices:

(21) $$J\left(X_{k}^{q}\right)=J_{P}\left(X_{k}^{q}\right)+J_{D}\left(X_{k}^{q}\right)$$

where $J_{P}\left(X_{k}^{q}\right)$ and $J_{D}\left(X_{k}^{q}\right)$ are the Fisher information matrix (FIM) of the priori information and the data, respectively.

(22) $$J_{P}\left(X_{k}^{q}\right)=E_{X_{k}^{q}}\left\{-\Delta_{X_{k}^{q}}^{X_{k}^{q}}\log p\left(X_{k}^{q}\right)\right\}$$

(23) $$J_{D}\left(X_{k}^{q}\right)=E_{X_{k}^{q},Z_{k}^{q}}\left\{-\Delta_{X_{k}^{q}}^{X_{k}^{q}}\log p\left(Z_{k}^{q}|X_{k}^{q}\right)\right\}$$

Combined with the system model in Section 2, the qth target is tracked by a fixed number of radars at the time index k. Since the radar independently observes the target at the same moment, the BIM of the target state can be simply expressed as:

(24) $$J\left(X_{k}^{q}\right)=J_{P}\left(X_{k}^{q}\right)+\sum_{i=1}^{N}u_{i,k}^{q}J_{D}^{\left(i\right)}\left(X_{k}^{q}\right)$$

where $J_{D}^{\left(i\right)}\left(X_{k}^{q}\right)$ is the FIM of the ith radar’s measurement on qth target. In (24), the term $J_{P}\left(X_{k}^{q}\right)$ can be calculated iteratively through the following formula:

(25) $$J_{P}\left(X_{k}^{q}\right)=D_{k-1}^{22}-D_{k-1}^{21}{\left(J\left(X_{k-1}^{q}\right)+D_{k-1}^{11}\right)}^{-1}D_{k-1}^{12}$$

where,

(26) $$D_{k-1}^{11}=E_{X_{k-1}^{q}X_{k}^{q}}\left\{-\Delta_{X_{k-1}^{q}}^{X_{k-1}^{q}}\log p\left(X_{k}^{q}|X_{k-1}^{q}\right)\right\}$$

(27) $$D_{k-1}^{12}=D_{k-1}^{21}=E_{X_{k-1}^{q}X_{k}^{q}}\left\{-\Delta_{X_{k}^{q}}^{X_{k-1}^{q}}\log p\left(X_{k}^{q}|X_{k-1}^{q}\right)\right\}$$

(28) $$D_{k-1}^{22}=E_{X_{k-1}^{q}X_{k}^{q}}\left\{-\Delta_{X_{k}^{q}}^{X_{k}^{q}}\log p\left(X_{k}^{q}|X_{k-1}^{q}\right)\right\}$$

Combined with the target dynamic model in Section 2.1, $J_{P}\left(X_{k}^{q}\right)$ can be written as:

(29) $$J_{P}\left(X_{k}^{q}\right)={\left[Q^{q}+FJ^{-1}\left(X_{k-1}^{q}\right)F^{T}\right]}^{-1}$$

For the ith radar, the FIM of the data can be given by:

(30) $$J_{D}^{\left(i\right)}\left(X_{k}^{q}\right)=E_{X_{k}^{q},Z_{i,k}^{q}}\left\{-\Delta_{X_{k}^{q}}^{X_{k}^{q}}\log p\left(Z_{i,k}^{q}|X_{k}^{q}\right)\right\}=E_{X_{k}^{q}}\left\{E_{Z_{i,k}^{q}|X_{k}^{q}}\left\{-\Delta_{X_{k}^{q}}^{X_{k}^{q}}\log p\left(Z_{i,k}^{q}|X_{k}^{q}\right)\right\}\right\}$$

According to [15], we can get:

(31) $$J_{D}^{\left(i\right)}\left(X_{k}^{q}\right)=E_{X_{k}^{q}}\left\{{\left(H_{i,k}^{q}\right)}^{T}{\left(G_{i,k}^{q}\right)}^{-1}H_{i,k}^{q}\right\}$$

where $H_{i,k}^{q}$ is the Jacobi matrix of $h_{i}\left(X_{k}^{q}\right)$ and can be expressed as:

(32) $$H_{i,k}^{q}={\left[\nabla_{X_{k}^{q}}{\left(h_{i}\left(X_{k}^{q}\right)\right)}^{T}\right]}^{T}=\left[\nabla_{X_{k}^{q}}R_{i,k}^{q},\nabla_{X_{k}^{q}}\theta_{i,k}^{q}\right]$$

where

(33) $$\nabla_{X_{k}^{q}}R_{i,k}^{q}={\left[\nabla_{x_{k}^{q}}R_{i,k}^{q},\nabla_{{\dot{x}}_{k}^{q}}R_{i,k}^{q},\nabla_{y_{k}^{q}}R_{i,k}^{q},\nabla_{{\dot{y}}_{k}^{q}}R_{i,k}^{q}\right]}^{T}$$

(34) $$\nabla_{X_{k}^{q}}\theta_{i,k}^{q}={\left[\nabla_{x_{k}^{q}}\theta_{i,k}^{q},\nabla_{{\dot{x}}_{k}^{q}}\theta_{i,k}^{q},\nabla_{y_{k}^{q}}\theta_{i,k}^{q},\nabla_{{\dot{y}}_{k}^{q}}\theta_{i,k}^{q}\right]}^{T}$$

are the first-order partial derivatives of the target distance and azimuth to the position and velocity, respectively.

Substitute (29) and (31) into (24), we can get the BIM of the target state $X_{k}^{q}$:

(35) $$J\left(X_{k}^{q}\right)={\left[Q^{q}+FJ^{-1}\left(X_{k-1}^{q}\right)F^{T}\right]}^{-1}+\sum_{i=1}^{N}u_{i,k}^{q}E_{X_{k}^{q}}\left\{{\left(H_{i,k}^{q}\right)}^{T}{\left(G_{i,k}^{q}\right)}^{-1}H_{i,k}^{q}\right\}$$

The first prior information FIM of $J\left(X_{k}^{q}\right)$ is only related to the BIM of the target state at the time index k−1 and the target dynamic model in Section 2.1. According to (7) and (8), the $G_{i,k}^{q}$ in the second item is related to the ith radar’s bandwidth on the qth target and the radar echo SNR at time index k. Meanwhile, SNR is a function of the dwell time. As a result, $J\left(X_{k}^{q}\right)$ is related to the bandwidth and the dwell time at time index k, thus laying the foundation for the joint dwell time and the bandwidth optimization strategy. Furthermore, in order to satisfy the demand of real-time, we can approximate (35) as:

(36) $$J\left(X_{k}^{q}\right)={\left[Q^{q}+FJ^{-1}\left(X_{k-1}^{q}\right)F^{T}\right]}^{-1}+\sum_{i=1}^{N}u_{i,k}^{q}{\left(H_{i,k}^{q}\right)}^{T}{\left(G_{i,k}^{q}\right)}^{-1}H_{i,k}^{q}$$

According to (18), the corresponding BCRLB matrix of the target state estimation error can be calculated as:

(37) $$C_{\mathrm{BCRLB},k}^{q}=J^{-1}\left(X_{k}^{q}\right)={\left[{\left[Q^{q}+FJ^{-1}\left(X_{k-1}^{q}\right)F^{T}\right]}^{-1}+\sum_{i=1}^{N}u_{i,k}^{q}{\left(H_{i,k}^{q}\right)}^{T}{\left(G_{i,k}^{q}\right)}^{-1}H_{i,k}^{q}\right]}^{-1}$$

### 3.2. Problem Formulation

This part our main task is to formulate the optimization problem, whose objective is minimizing the total dwell time of the radar network with the tracking performance meeting a predefined threshold.

In Section 3.1, we derived the BCRLB of the target tracking error, which can be used to measure the target tracking accuracy. Moreover, given the updated BIM $J\left(X_{k-1}^{q}\right)$ at the time index k−1 and the radar radiation parameters, we can now determine the predictive BCRLB of the target q at time index k according to the formula (37):

(38) $$C_{\mathrm{BCRLB},k|k-1}^{q}={\left[{\left[Q^{q}+FJ^{-1}\left(X_{k-1}^{q}\right)F^{T}\right]}^{-1}+\sum_{i=1}^{N}u_{i,k}^{q}{\left(H_{i,k|k-1}^{q}\right)}^{T}{\left(G_{i,k|k-1}^{q}\right)}^{-1}H_{i,k|k-1}^{q}\right]}^{-1}$$

where $G_{i,k|k-1}^{q}$ and $H_{i,k|k-1}^{q}$ are the predicted values of $G_{i,k}^{q}$ and $H_{i,k}^{q}$, respectively. The diagonal element of $C_{\mathrm{BCRLB},k|k-1}^{q}$ is the lower bound of the estimated MMSE of the target state estimation, which can be extracted as a measurement metric of target tracking accuracy:

(39) $$F_{k|k-1}^{q}=\sqrt{C_{k|k-1}^{q}\left(1,1\right)+C_{k|k-1}^{q}\left(3,3\right)}$$

where $C_{k|k-1}^{q}\left(1,1\right)$ and $C_{k|k-1}^{q}\left(3,3\right)$ are the first variable and the third variable on the diagonal $C_{k|k-1}^{q}$, respectively.

Since the tracking accuracy meets a predefined threshold $F_{\max}$, the constraint on the accuracy is:

(40) $$F_{k|k-1}^{q}\leq F_{\max},\;\forall q=1,2,\ldots ,Q$$

Then, with respect to the total bandwidth budget, if $u_{i,k}^{q}=1$, the bandwidth of the ith radar’s illumination on the qth target at time index k should satisfy an upper bound $\beta_{\max}$ and a lower bound $\beta_{\min}$:

(41) $$\{\begin{array}{ll} \beta_{i,q,k}=0, & u_{i,k}^{q}=0\\ \beta_{\min}\leq \beta_{i,q,k}\leq \beta_{\max},\; & u_{i,k}^{q}=1 \end{array}$$

Similarly, the dwell time constraints can be denoted as:

(42) $$\{\begin{array}{ll} T_{i,q,k}^{d}=0, & u_{i,k}^{q}=0\\ T_{\min}^{d}\leq T_{i,q,k}^{d}\leq T_{\max}^{d},\; & u_{i,k}^{q}=1 \end{array}$$

We define the data processing rate of fusion center as ε, and the total number of samples in the radar network should satisfy the following constraints:

(43) $$\sum_{q=1}^{Q}N_{k}=\frac{1}{\varepsilon }$$

By fusing (40), (41), (42) and (43) together, we can formulate the optimization problem for the joint dwell time and bandwidth optimization strategy:

(44) $$\begin{array}{c} \underset{T_{m,q,k}^{d}\beta_{i,k,q}}{\min}\sum_{q=1}^{Q}\sum_{i=1}^{N}T_{m,q,k}^{d}\\ s.t.\{\begin{array}{ll} F_{k|k-1}^{q}\leq F_{\max},\;\;\;\;\forall q=1,2,\ldots ,Q\\ \{\begin{array}{cc} \beta_{i,q,k}=0, & u_{i,k}^{q}=0\\ \beta_{\min}\leq \beta_{i,q,k}\leq \beta_{\max},\; & u_{i,k}^{q}=1 \end{array}\\ \{\begin{array}{cc} T_{i,q,k}^{d}=0, & u_{i,k}^{q}=0\\ T_{\min}^{d}\leq T_{i,q,k}^{d}\leq T_{\max}^{d},\; & u_{i,k}^{q}=1 \end{array}\\ \sum_{q=1}^{Q}N_{k}=\frac{1}{\varepsilon },\;\sum_{q=1}^{Q}u_{i,k}^{q}\leq 1,\; & \sum_{m=1}^{N}u_{i,k}^{q}=M \end{array} \end{array}$$

where $\sum_{q=1}^{Q}u_{i,k}^{q}\leq 1$ represents that a single radar tracks at most one target in a revisit period. The term $\sum_{m=1}^{N}u_{i,k}^{q}=M$ represents that each radar is tracked by M radars at time index k. 

Since $u_{i,k}^{q}\in \left\{0,1\right\}$ is a binary variable, the optimization problem described in (44) is a non-convex problem with three parameters: radar selection, dwell time and the transmitted signal’s bandwidth. However, for a given $u_{k}^{q}$, assuming that the radar i is assigned to qth target, the unique radar node selection scheme for the qth can be determined. Furthermore, in order to ensure that all targets have enough information, assuming that each target has the same amount of samples which needs to be sent to the fusion center, then the optimization problem can be converted to the following formula, which only has the variables $T_{m,q,k}^{d}$ and $\beta_{m,q,k}$ (1≤m≤M):

(45) $$\begin{array}{l} \underset{T_{m,q,k}^{d}\beta_{i,k,q}}{\min}\sum_{m=1}^{M}T_{m,q,k}^{d}\\ s.t.\{\begin{array}{l} F_{k|k-1}^{q}\leq F_{\max}\\ \sum_{m=1}^{M}\beta_{m,q,k}=\frac{c}{Q\rho \varepsilon V}=\beta_{total}\\ \beta_{\min}\leq \beta_{i,q,k}\leq \beta_{\max}\\ T_{\min}^{d}\leq T_{i,q,k}^{d}\leq T_{\max}^{d} \end{array} \end{array}$$

where $\beta_{\mathrm{total}}$ is the total bandwidth of the transmitted waveform of all radars that are assigned to the same target.

### 3.3. Joint Dwell Time and Bandwidth Optimization Problem Solution

**Figure 1:**
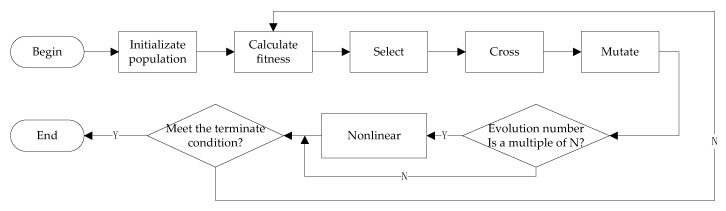
The nonlinear programming-based genetic algorithm (NPGA) flowchart.

The optimization problem proposed in Equation (45) is non-convex, containing two parameters $T_{m,q,k}^{d}$ and $\beta_{m,q,k}$. We can use the exhaustive method to solve it, which is simple but too inefficient. The genetic algorithm uses selection, cross and mutation operators for searching, which has a great global search ability. However, the local search ability of this genetic algorithm is weak. In contrast, most of the classical nonlinear algorithms adopt the means of the gradient method, which has a strong local search ability, while also possessing a weak global search ability. As a result, we will solve the problem in (45) by NPGA [29], which combines the global search ability of the genetic algorithm and the local search ability of the classical nonlinear programming algorithms. The flowchart of NPGA is shown in Figure 1:

By working out the problem (45) for $Q\cdot C_{N}^{M}$ times, we can get all the optimal solutions of the dwell time with respect to different target and radar combinations in the constraint of $\sum_{i=1}^{N}u_{i,k}^{q}=M$. Then we can use the exhaustive method to obtain the optimal results of the dwell time and radar allocation index in the constraints of $\sum_{q=1}^{Q}u_{i,k}^{q}\leq 1$. However, the exhaustive method is complex and inefficient. As a result, we propose a radar node selection algorithm with lower computation complexity.

Assuming M=2, which means that each target is fixed to be tracked by two radars at each moment. We define $R_{l}=\left\{a,b\right\}\left(l=1,2,\ldots ,L\right)$ as the combinations of the two radars in the radar network, where $L=C_{N}^{2}=\frac{N!}{\left(N-2\right)!2!}$. When the target q is illuminated by the $R_{l}$ index radars, suppose $S_{l,k,q,\min}={\left(T_{a,q,k,\min}^{d}\right)}^{\left(l\right)}+{\left(T_{b,q,k,\min}^{d}\right)}^{\left(l\right)}$ denotes the minimum dwell time which is solved in (45) through NPGA, where ${\left(T_{a,q,k,\min}^{d}\right)}^{\left(l\right)}$ and ${\left(T_{b,q,k,\min}^{d}\right)}^{\left(l\right)}$ denotes the dwell times of radar a and radar b, respectively. The minimum dwell time matrix $S_{k,\min}$ which is composed of $S_{l,k,q,\min}$, is shown in Table 1.

Similar to the term $u_{i,k}^{q}$, we define a set of binary variables $U_{l,k}^{q}\in \left\{0,1\right\}$ to represent the radar combination selection index.

(46) $$U_{l,k}^{q}=\{\begin{array}{l} 1,\;\mathrm{if}\;\mathrm{the}\;q\mathrm{th}\;\mathrm{radar}\;\mathrm{is}\;\mathrm{tracked}\;\mathrm{by}\;\mathrm{the}\;l\mathrm{th}\;\mathrm{radar}\;\mathrm{combination}\;\mathrm{at}\;\mathrm{time}\;\mathrm{index}\;k\\ 0,\;\mathrm{otherwise} \end{array}$$

Then the optimization model of the radar combination allocation index can be described as:

(47) $$\begin{array}{l} \min\sum_{q=1}^{Q}\sum_{l=1}^{L}U_{l,k}^{q}S_{l,q,k,\min}\\ s.t.\left\{ \begin{array}{l} \;\sum_{l=1}^{L}U_{l,k}^{q}=1\\ \left(\overset{L}{\underset{l=1}{\cup }}U_{l,k}^{r}R_{l}\right)\cap \left(\overset{L}{\underset{l=1}{\cup }}U_{l,k}^{m}R_{l}\right)=\emptyset ,\forall r\neq m,r,m=1,2,\ldots ,Q \end{array}\right. \end{array}$$

where the first constraints imply that each target is tracked by a fixed radar combination at time index k, while the second one suggests that a single radar tracks at most one target at k. The solution method of (47) can be shown in Algorithm 1.
**Algorithm 1.** Radar allocation method**Step (1):** Working out the problem in (47) $Q\cdot \frac{N!}{\left(N-2\right)!2!}$ times, then we can get the minimum dwell time matrix $S_{k,\min}$ in the constraint of $\sum_{i=1}^{N}u_{i,k}^{q}=2$. **Step (2):** Sort the columns of matrix $S_{k,\min}$ in ascending order and assign the target corresponding to the smallest element in the first row to the corresponding radar combination. **Step (3):** Remove the column vectors corresponding to the target assigned in Step (2). Remove all the row vectors of the radar which is contained in the radar combination assigned in Step (2). **Step (4):** Repeat Step (2) and Step (3) until all the targets are assigned in order to obtain the optimal allocation matrix $U_{k,\mathrm{opt}}$.

By using the above algorithm, we can obtain the optimal radar allocation results $U_{k,\mathrm{opt}}$, where $U_{k,\mathrm{opt}}=\left[U_{k,\mathrm{opt}}^{1},U_{k,\mathrm{opt}}^{2},\ldots ,U_{k,\mathrm{opt}}^{Q}\right]$, $U_{k,\mathrm{opt}}^{q}={\left[U_{1,k,\mathrm{opt}}^{q},U_{2,k,\mathrm{opt}}^{q},\ldots ,U_{L,k,\mathrm{opt}}^{q}\right]}^{T}$. When $U_{l,k,\mathrm{opt}}^{q}=1$, $u_{a,k,\mathrm{opt}}^{q}=u_{b,k,\mathrm{opt}}^{q}=1$, $T_{a,q,k,\mathrm{opt}}^{d}={\left(T_{a,q,k,\min}^{d}\right)}^{\left(l\right)}$, $T_{b,q,k,\mathrm{opt}}^{d}={\left(T_{b,q,k,\min}^{d}\right)}^{\left(l\right)}$. When $U_{l,k,\mathrm{opt}}^{q}=0$, $u_{a,k,\mathrm{opt}}^{q}=u_{b,k,\mathrm{opt}}^{q}=0$, $T_{a,q,k,\mathrm{opt}}^{d}=T_{b,q,k,\mathrm{opt}}^{d}=0$. Then we can get $u_{k,\mathrm{opt}}$ and $T_{k,\mathrm{opt}}^{d}$ at time index k, which are the radar allocation index and dwell time optimization results, respectively.

The computational complexity of 0 is $O\left(\frac{Q^{2}}{2}\times \frac{N!}{\left(N-2\right)!2!}\log_{2}\left(\frac{N!}{\left(N-2\right)!2!}\right)\right)$, while the computational complexity of the exhaustive method is $O\left({\left(\frac{N!}{\left(N-2\right)!2!}\right)}^{Q}\right)$. Compared with the enumeration method, 0 can greatly reduce the computational complexity and improve the real-time performance.

## 4. Simulation Results

In this section, some numerical results are provided to illustrate the performance of the proposed LPI-based joint dwell time and bandwidth optimization strategy for multi-target tracking in a radar network. A multi-target tracking scenario with six radars and two targets is considered. In order to simplify the problem, we assume that all the radars in the network systems have the same system parameters. Then we can utilize the default values for the system parameters, as given in Table 2.

The velocities of target 1 and target 2 are: (1300,530)m/s and (−1300,−530)m/s, respectively. It is also assumed that the tracking process lasts 150 s.

**Figure 2:**
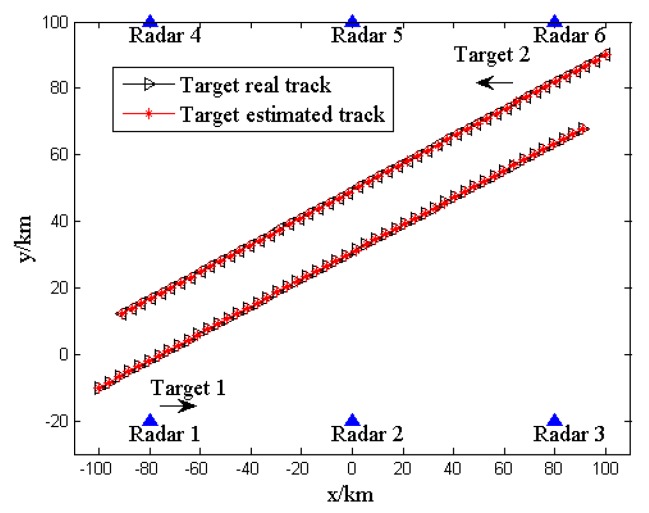
Target trajectory and radar network deployment.

Figure 2 depicts the distribution of the radar network, the true trajectories of the two targets and the estimated trajectories of the targets according to the proposed strategy.

This part first gives the simulation results under the non-undulating RCS model. Assuming that the reflection coefficients of all targets is 1 at any observation time, we define this situation as RCS case 1. In this case, the radar selection and dwell time allocation are only related to the distance and relative position of the target to the radar.

**Figure 3:**
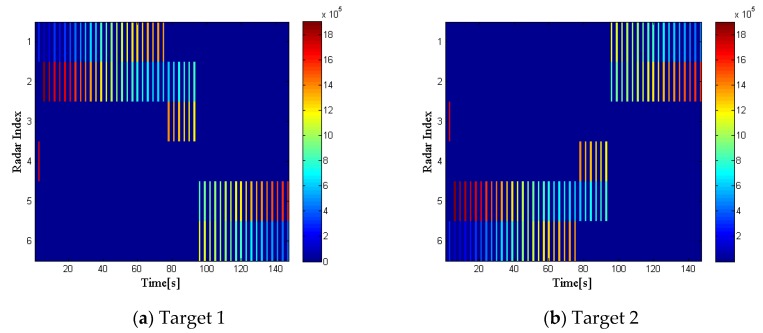
Radar selection and bandwidth allocation in radar cross section (RCS) case 1.

**Figure 4:**
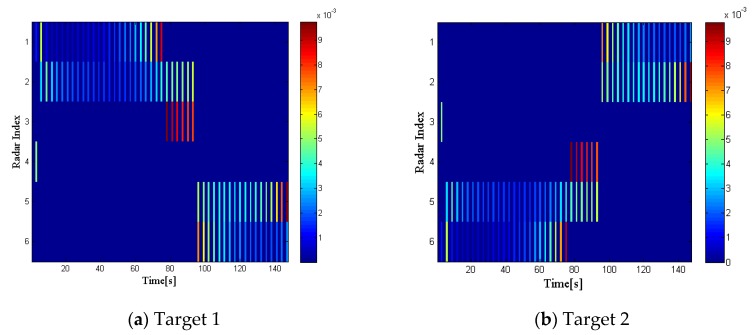
Radar selection and dwell time allocation in RCS case 1.

Figure 3 shows the radar selection and bandwidth allocation results of the two targets, while Figure 4 gives the dwell time allocation results. In each figure, on the left side is the radar index, on the right side is the different intensity of the bandwidth and dwell time, which is depicted in different colors. Moreover, the blue areas in each figure indicate that the radar selection variable $u_{i,k}^{q}=0$, while the lines in different colors mean that $u_{i,k}^{q}=1$, with different colors representing the intensity of the transmitted bandwidth and dwell time. We can conclude that the radar network tends to assign the two radars closest to a specified target for tracking tasks, and more dwell time and bandwidth resources will be allocated to the selected radar, which is farther from the target.

**Figure 5:**
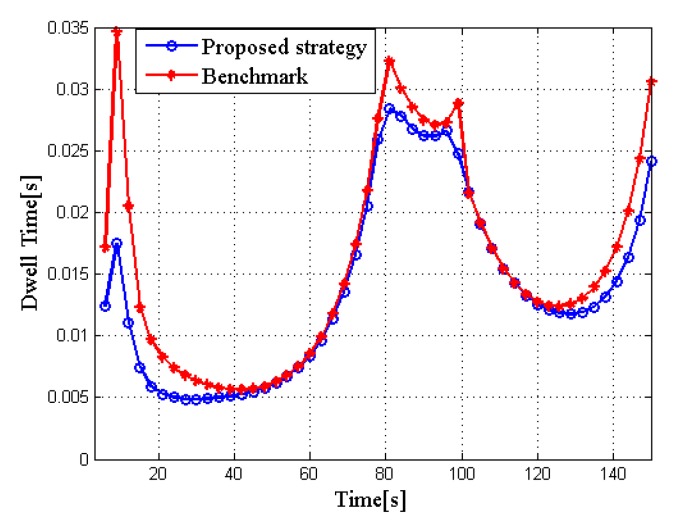
Comparison of total dwell time for different algorithms in RCS case 1.

To show the superiority of the proposed joint dwell time and bandwidth optimization strategy, the optimization algorithm without considering the bandwidth allocation is compared to a benchmark. Figure 5 shows the comparison of total dwell time for two different algorithms. From the result we can see that the proposed strategy can reduce the total dwell time of the radar network compared with the benchmark.

Define the root mean square error (RMSE) for the tracking accuracy of all targets at time index k as:

(48) $$\mathrm{RMSE}\left(k\right)=\sum_{q=1}^{Q}\sqrt{\frac{1}{N_{\mathrm{MC}}}\sum_{n=1}^{N_{\mathrm{MC}}}\left\{{\left[x_{k}^{q}-{\hat{x}}_{n,k|k}^{q}\right]}^{2}+{\left[y_{k}^{q}-{\hat{y}}_{n,k|k}^{q}\right]}^{2}\right\}}$$

where $N_{\mathrm{MC}}=100$ represents the Monte Carlo experiment number, and $\left({\hat{x}}_{n,k|k}^{q},{\hat{y}}_{n,k|k}^{q}\right)$ is the location estimate at the nth trial.

**Figure 6:**
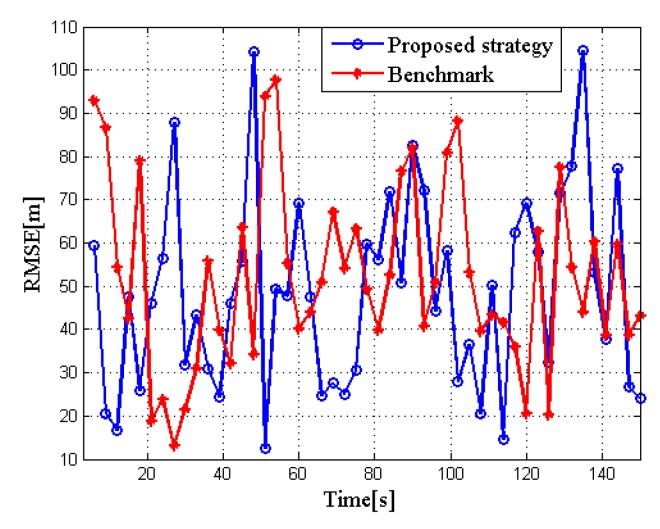
Root mean squared error (RMSE) in two algorithms for target tracking in RCS case 1.

The RMSE of the proposed strategy and the benchmark are evaluated in Figure 6, respectively. The results prove that the tracking accuracy has not been sacrificed too much after allocating the bandwidth, which is acceptable to our tracking tasks.

**Figure 7:**
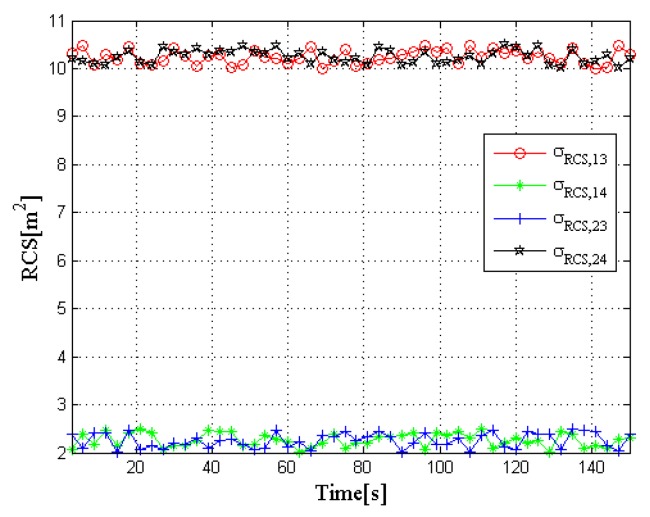
RCS case 2.

In order to further analyze the impact of the target RCS on radar selection and radar resource allocation results, a second RCS model is also considered, which can be defined as RCS case 2, where it is depicted in Figure 7. In this case, the reflection coefficient of the two targets to radar 3 and radar 4 change with time, while the RCS of the two targets to the other radars remain unchanged at any observation time. In Figure 7, the red and black lines represent the RCS values of target 1 to radar 3 and target 2 to radar 4 at each moment, respectively, which fluctuate around 10.3 m^2^. Similarly, the green and blue lines represent the RCS values of target 1 to radar 4 and target 2 to radar 3, respectively, which fluctuate around 2.3 m^2^.

**Figure 8:**
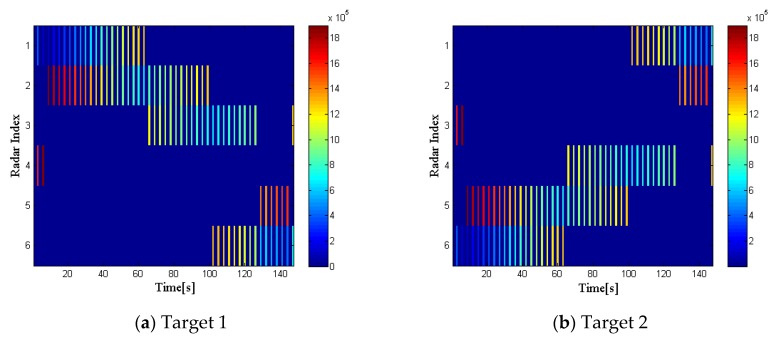
Radar selection and bandwidth allocation in RCS case 2.

**Figure 9:**
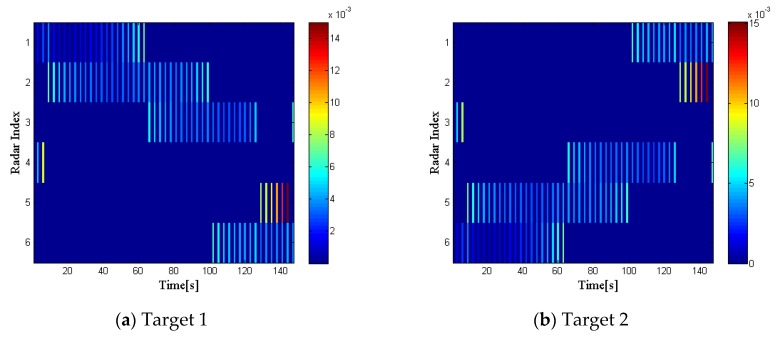
Radar selection and bandwidth allocation in RCS case 2.

Figure 8 and Figure 9 illustrate the optimization results of target 1 and target 2 with the proposed strategy in RCS case 2 at every time index, respectively.

Compared with Figure 3 and Figure 4, we can draw the following conclusions. During the whole tracking process, the number of times that radar 3 irradiated target 1 and radar 4 irradiated target 2 increase significantly. In addition, during the period in which radar 2 and radar 3 irradiate target 1 together, radar 2, which is closer to the target, but has a lower reflection coefficient, is allocated more bandwidth and dwell time resources. Similarly, this phenomenon also exists in the resource allocation of target 2. In summary, it can be concluded that the reflection coefficient of the target also affects the radar selection and radar resource allocation results. The radar network system will preferentially select the radar with higher reflection coefficient to irradiate the target. Furthermore, the system tends to allocate more resources to the radar with lower reflection coefficient to the target.

**Figure 10:**
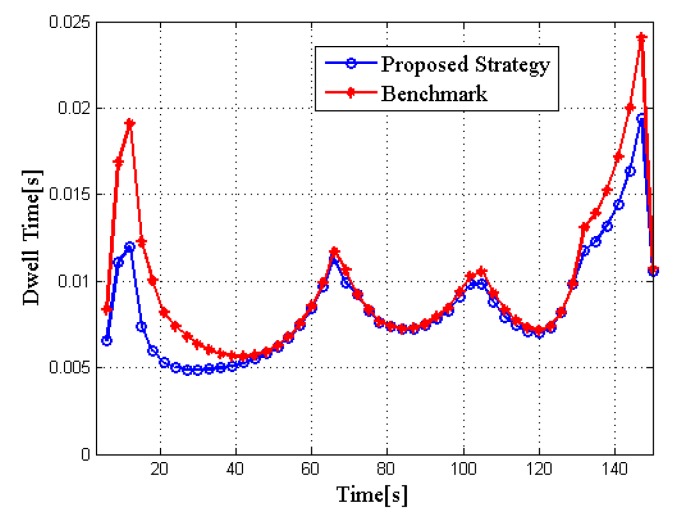
Comparison of total dwell time for different algorithms in RCS case 2.

**Figure 11:**
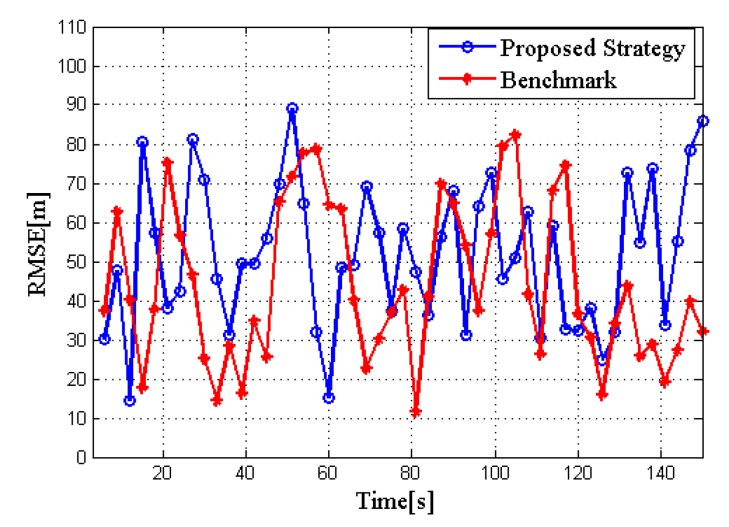
RMSE in two algorithms for target tracking in RCS case 2.

Figure 10 and Figure 11 show the performance comparison of the two algorithms in RCS case 2. Obviously, it is consistent with the conclusions of RCS case 1, thus verifying the stability of the proposed strategy.

**Figure 12:**
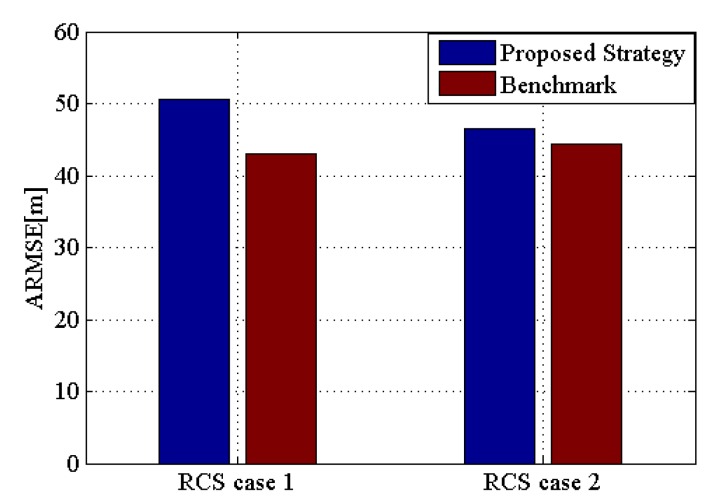
Average root mean square error (ARMSE) comparison of two algorithms for target tracking.

Define the target tracking average root mean square error (ARMSE) as:

(49) $$\mathrm{ARMSE}\left(k\right)=\sum_{q=1}^{Q}\sqrt{\frac{1}{N_{\mathrm{MC}}}\sum_{n=1}^{N_{\mathrm{MC}}}\frac{1}{N_{k}^{q}\left(n\right)}\sum_{k=1}^{N_{k}^{q}\left(n\right)}\left\{{\left[x_{k}^{q}-{\hat{x}}_{n,k|k}^{q}\right]}^{2}+{\left[y_{k}^{q}-{\hat{y}}_{n,k|k}^{q}\right]}^{2}\right\}}$$

where $N_{k}^{q}\left(n\right)$ denotes the number of times that the radar network radiated qth target at time index k. Figure 12 shows the ARMSE comparison between the proposed strategy and the benchmark in the two RCS cases. With respect to the target tracking accuracy, the latter is slightly better than the former, but the gap is not large, and is within an acceptable range. In conclusion, the proposed strategy effectively improves the LPI performance of the radar network without sacrificing too much accuracy.

## 5. Conclusions

An LPI-based joint dwell time and bandwidth allocation strategy is proposed in this paper. The basis of this strategy is to use the optimization technique to control the radars’ illumination in the radar network for the purpose of improving the LPI performance. Meanwhile, the tracking accuracy of each target must be guaranteed, which means that the BCRLB meets a predefined threshold. The physical explanation of this strategy can be described as: (1) For each target, select a suitable radar group to complete tracking tasks; (2) Under the premise of tracking tasks requirements, minimize the total dwell time of radar network. The resulting optimization problem contains two adaptable vectors, one for dwell time and the other for bandwidth allocation, which is solved by NPGA, and then a proposed algorithm. Simulation results demonstrate that the proposed strategy can achieve a better LPI performance compared with the benchmark.

In future work, more illumination resources, such as the transmitted power of each radar, will be taken into consideration. Furthermore, the cases of detection probability less than 1 and false alarm probability greater than 0 are of practical importance, which should be taken into account [27,28].

## Figures and Tables

**Table 1:** Minimum dwell time matrix for the fixed radar combination (M=2).

The Minimum Dwell Time of Different Radar Combination		Target			
		1	2	…	*Q*
**Radar** **Combination**	$R_{1}=\left\{1,2\right\}$	$S_{1,1,k,\min}$	$S_{1,2,k,\min}$	…	$S_{1,Q,k,\min}$
	$R_{2}=\left\{1,3\right\}$	$S_{2,1,k,\min}$	$S_{2,2,k,\min}$	…	$S_{2,Q,k,\min}$
	⋮	⋮	⋮	⋮	⋮
	$R_{L}=\left\{N-1,N\right\}$	$S_{L,1,k,\min}$	$S_{L,2,k,\min}$	…	$S_{L,Q,k,\min}$

**Table 2:** Radar network system parameters.

Parameter	Value	Parameter	Value
$P_{t}$	500 W	$\sigma^{q}$	$1{\;m}^{2}$
λ	0.03 m	$T_{r}$	$5\times 10^{-4}\;s$
$\beta_{\mathrm{total}}$	2 MHz	$F_{\max}$	30 m
$\beta_{\min}$	0.1 MHz	$\beta_{\max}$	1.9 MHz
$\theta_{3\mathrm{dB}}$	$2^{o}$	γ	$1{\;m}^{2}$
$T_{\min}^{d}$	$5\times 10^{-4}\;s$	$T_{\max}^{d}$	0.1 s
